# Patient-derived organoids (PDOs) and PDO-derived xenografts (PDOXs): New opportunities in establishing faithful pre-clinical cancer models

**DOI:** 10.1016/j.jncc.2022.10.001

**Published:** 2022-10-22

**Authors:** Ergang Wang, Kun Xiang, Yun Zhang, Xiao-Fan Wang

**Affiliations:** 1 Department of Pharmacology and Cancer Biology, Duke University, Durham, United States; 2 State Key Laboratory of Molecular Oncology, National Cancer Center/National Clinical Research Center for Cancer/Cancer Hospital, Chinese Academy of Medical Sciences and Peking Union Medical College, Beijing, China

**Keywords:** Patient-derived organoid, Patient-derived xenograft, PDO-derived xenograft

## Abstract

One of the major bottlenecks in advancing basic cancer research and developing novel cancer therapies is the lack of *in vitro* pre-clinical models that faithfully recapitulate tumor properties in the patients. Monolayer cultures of cancer cell lines usually lose the heterogeneity of the parental tumors, while patient-derived xenograft (PDX) suffers from its time- and resource-intensive nature. The emergence of organoid culture system and its application in cancer research provides a unique opportunity to develop novel *in vitro* cancer pre-clinical models. Here we review the recent advances in utilizing organoids culture system and other related three-dimensional culture systems in studying cancer biology, performing drug screening, and developing cancer therapies. In particular, we discuss the advantages of applying xenograft initiated from patient-derived organoids (PDOs) as a faithful cancer pre-clinical model in basic cancer research and precision medicine.

## 1 Introduction

Cancer remains one of the major health problems worldwide despite the ever-increasing knowledge and investments in cancer related research and therapeutic development. One of the major bottlenecks is the lack of 3D pre-clinical models of cancer that can faithfully recapitulate tumor properties to study the disease progression and develop novel treatments. For decades, the most widely applied cancer models are transgenic mouse models and monolayer culture of cancer cell lines. Although important insights of cancer biology have been gained through these models, both experimental systems have obvious drawbacks. For example, genetically modified mouse models of cancer are in general time-consuming and may not fully recapitulate the disease progression of cancer patients, particularly for certain features such as genetic heterogeneity and histological complexity.^1^ On the other hand, the *in vitro* 2D culture models exert selective pressure on cancer cells, and thus only a handful of rapidly proliferating cancer cell clones may expand from tumor samples derived from human patients. Furthermore, the 2D cultured cancer cell lines gradually lose the heterogeneity of the parental tumors as they are passaged. Due to all these limitations of conventional cancer models, therapeutic treatments developed from these models often fail to be translated into clinical settings, suggesting that these are not reliable pre-clinical models.

Patient-derived models of cancer (PDMC), such as patient-derived xenografts (PDXs) and patient-derived organoids (PDOs), have been developed aiming to address the limitations of the conventional models. Both PDO and PDX capture the clinical heterogeneity of cancer in terms of stage, molecular features, and genetic background. Consequently, they can better represent the original patient tumors.^2^ Indeed, both PDO and PDX were found to preserve the genetic mutations of the original tumor, and initial drug sensitivity tests on both models have demonstrated their abilities to faithfully recapitulate the patient-specific responses to chemotherapies and targeted therapies.^3^, ^4^, ^5^, ^6^ Therefore, PDO and PDX are becoming the gold standards in pre-clinical therapeutic development.

PDX is an *in vivo* model by implanting human patient tumor materials into immunodeficient mice,^7^^,^^8^ where they develop into xenografts in the recipient animals. The patient tumor materials can be either minced tissues or single-cell suspensions. The most common form of implantation is via subcutaneous injections, while other approaches including orthotopic injection are also widely performed.^8^ The PDX model is not a new concept, with some initial attempts that could be traced back to the 1980s.^9^^,^^10^ These early studies demonstrated that PDX models and their corresponding patients showed similar responses to certain therapeutic treatments. The more recent clinical needs for developing new therapeutic options for cancer have brought the PDX model back to the spotlight. PDX can be established from many types of cancer, including colorectal cancer (CRC),^11^^,^^12^ breast cancer,^13^, ^14^, ^15^ pancreatic ductal adenocarcinoma (PDAC),^16^, ^17^, ^18^ and others.^19^, ^20^, ^21^ Compared to 2D cultured cancer cell lines, PDX is rich in stromal component, which may be beneficial for studies focusing on the interactions between cancer cell and the tumor microenvironment (TME). The global gene-expression patterns, mutational status, metastatic potentials, and histopathology characteristics of the human donor tumor have been shown to be well-maintained in PDX.^7^^,^^22^ Therefore, it can be applied to develop personalized cancer treatment.^23^ However, there are certain limitations in utilizing PDX, for example, the high variation in successful engraftment rates among different types of cancer (Table 1). For instance, the engraftment rate from surgical resections of CRC could be around 60∼80%,^4^^,^^11^ while only around 20% in breast cancer PDXs.^7^^,^^13^, ^14^, ^15^ For multiple cancer types, the engraftment rate is even lower for deriving PDX using fine-needle biopsy (FNB) (0–36.4%) compared with those using surgical specimens (27.3%−70%).^24^ Therefore, the success in the engraftment of PDX strongly depends on the amount of starting tumor material so that surgical specimens are the most commonly used original source.^7^ Moreover, PDX requires a long engraftment period, typically from four to eight months. Thus, it is often challenging to provide timely drug screening results for the patients by using the PDX model. In addition, PDX suffers from low efficiency of cryopreservation and subsequent reanimation, which may lead to loss of irreplaceable tumor samples.^25^ The inevitable long-term nature of housing mice for PDX is cost-prohibitive, which in turn discourages the use of PDX for high-throughput screenings.

**Table 1:** Summary of PDX success rate in different cancer types.

	Uptake rate	References
**Colorectal cancer**	54/85 (63.5%); 35/40 (87.5%)	Julien et al.^11^; Puig et al.^12^
**Breast cancer**	25/200 (12.5%); 18/49 (37%); 37/158 (23.4%)	Marangoni et al.^13^; DeRose et al.^14^; Vaillant et al.^15^
**PDAC**	44/62 (71%); 42/69 (61%); 10/16 (62%)	Moffitt et al.^16^; Garrido-Laguana et al.^17^; Reyes et al.^18^
**Prostate cancer**	26/261 (10%)	Nguyen et al.^19^
**Bladder cancer**	22/54 (41%)	Pan et al.^20^
**Upper tract urothelial carcinoma**	17/34 (50%)	Kim et al.^21^

Abbreviations: PDAC, pancreatic ductal adenocarcinoma; PDX, patient-derived xenograft.

Unlike xenografts, organoid is an *in vitro* 3D culture model. The pioneering work of culturing 3D tissue structures was done in the 1980s and 1990s by James Rheinwald and Howard Green.^26^, ^27^, ^28^ In 2009, Hans Clevers's lab demonstrated that epithelial organoids could be established from a single LGR5^+^ intestinal stem cell.^29^ Since then, organoids from many organs and tissues have been developed. The current definition of organoids is “a 3D structure grown from stem cells and consisting of organ-specific cell types that self-organizes through cell sorting and spatially restricted lineage commitment”.^28^ Two types of stem cells are usually utilized to develop organoids: pluripotent stem cells (PSC)/induced pluripotent stem cells (iPSC), which initiate a range of tissues or organs induced by different media, and adult stem cells (ASC), which represent the tissues or organs they originally reside.

The organoid culture system can also be extended into cancer research, the first attempt of which was to culture colon adenocarcinoma organoids from *Apc*-deficient mice.^30^ Compelling evidence indicated that PDOs can be derived by directly embedding the patient tumor cells or tissues into the laminin-rich extracellular matrix - Matrigel and can be cultured under the serum-free conditional medium while preserving the characteristics of the parental tumor.^31^ Other methods, like air-liquid interface (ALI) organoids^32^ and micro-organospheres,^33^ are also widely used to develop PDO for conducting basic research and screening for effective therapies. Similar to PDX, PDO biobanks have been established for many types of cancer, such as colorectal,^34^, ^35^, ^36^, ^37^, ^38^ pancreatic,^39^ hepatic,^40^ gastric,^41^^,^^42^ prostate,^43^ breast,^44^ and ovarian cancer,^45^^,^^46^ and specific PDO can be acquired from centralized organizations, for example the Human Cancer Models Initiative (HCMI). The fast-growing collection and storage of PDOs as biobanks have facilitated basic cancer research and clinical trials, substantially increasing the options for modeling different types of cancer.

**Fig. 1:**
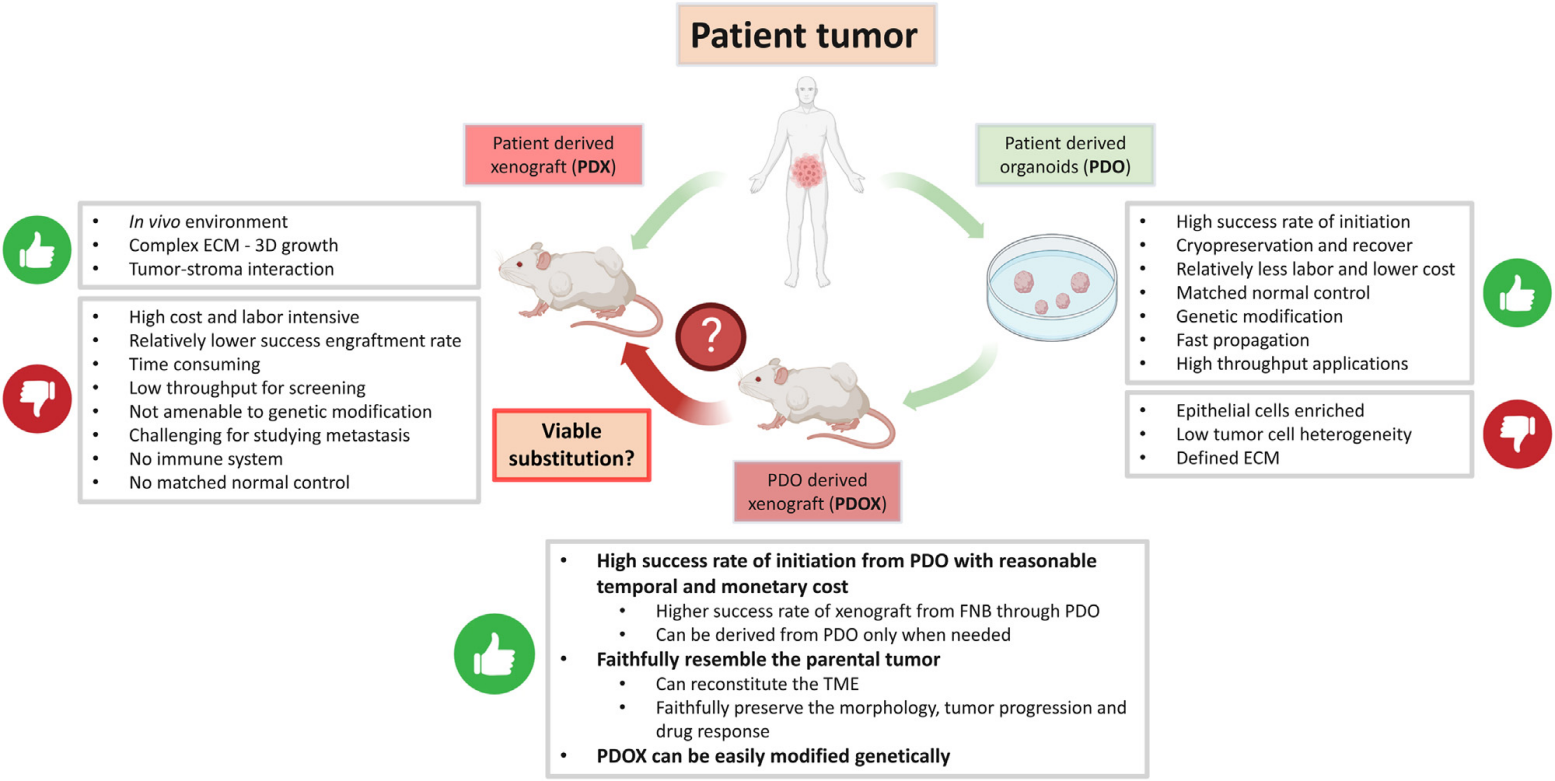
Comparison of patient-derived organoids (PDO), patient-derived xenografts (PDX) and PDO-derived xenografts (PDOX), along with a potential workflow for deriving patient-derived models of cancer (PDMC) in the future.

One of the advantages that PDX holds over conventional PDO is the presence of *in vivo* stromal component, which allows for studying the tumor-stroma interaction albeit the apparent differences between mouse and human patient stroma. However, the longstanding drawbacks of PDX limit its applications in precision medicine and tumor biobanking. In contrast, since PDO is an *in vitro* culture system, it can be established from patient tumor samples with a much higher success rate. It is also less expensive, requires less labor and propagates faster than PDX. Therefore, PDO permits high-throughput drug screening for patient tumors with significantly reduced cost and time. Moreover, there are significant limitations in applying PDX models in immuno-oncology studies since the xenografts are commonly inoculated into immune-compromised mice. Although recent progress in generating humanized mice have enabled the reconstitution of human immune cells in mouse models using human CD34^+^ cells or peripheral blood mononuclear cells (PBMC), certain limitations persist when using PDXs in such humanized mouse models to study immuno-oncology, including the HLA matching issue and the low fraction of CD4/8 ^+^ T cell populations.^47^^,^^48^ In this regard, while the traditional 3D submerged PDO selectively enriches for tumor cells, numerous newly developed PDO models have started to incorporate TME into the experimental setting, such as putting the immune cells and cancer-associated fibroblasts (CAF) into culture. These new models can accurately predict responses to chemotherapies and immunotherapies in their corresponding patients.^49^, ^50^, ^51^ Notably, organoids can be used to test unwanted side effects in order to prevent adverse drug reactions and determine the appropriate dosage of chemotherapy. Organoids have been deployed to assess drug toxicity in many organs.^52^ The establishment of normal and tumor tissue-derived organoid from the same patient enables personalized toxicity control in drug screening, and therefore provide optimal therapeutic guidance for the patients.^53^, ^54^, ^55^ Furthermore, xenografts derived from PDO (PDOX) have been successfully established. Similar to PDX, PDOX have been demonstrated to retain key pathological features of the parental tumor, such as the mutational profiles and the level of tumor heterogeneity.^56^^,^^57^ The establishment of PDOX grants more applicability to PDO and may overcome the limitations of both PDX and PDO (Fig. 1).

In this review, we will summarize recent advancements in PDO and PDO-derived culture systems, including immune cell and CAF co-culture systems, ALI systems and microfluidic murine- and patient-derived organotypic tumor spheroid (MDOT/PDOT) systems, as well as their applications in cancer research and precision medicine. In particular, this review will discuss the potential of PDOX as a new frontier of establishing faithful pre-clinical cancer models.

## 2 Developing PDO for different cancer types

PDO have been established for many different types of cancer (Table 2). One of the most widely established and studied cancer PDOs is the CRC organoid. In 2015, a biobank of 26 (22+4) CRC organoids was established from resected colon segments with a success rate of 90%.^34^ These organoids could be readily expanded and cryopreserved, with the typical survival rate after thawing to be > 80%, representing a significant advantage compared with PDX models. Shortly afterwards, CRC PDOs were successfully established from 18-gauge biopsies of metastatic lesions with a success rate > 70%. Importantly, these PDOs preserved genetic diversity of their parental metastases.^37^ In addition, niche factors that were often required for normal human colonic organoid culture, including epithelial growth factor (EGF), Noggin, TGF-β inhibitor A83–01, and p38 inhibitor SB202190 (ENAS), as well as Wnt3A/R-spondin1 and hypoxia, were carefully examined in the culture of CRC organoids.^35^ The niche dependency of CRC organoids indicated that CRC tissues largely retain the requirements of stem cell homeostasis in the normal intestinal epithelium. Decreased niche factor requirements in CRC organoids were observed in the transition from adenoma to carcinoma, reflecting the accumulation of genetic mutations in this process. It is worth noting that by establishing eight combinatorial culture conditions with different niche factors, a 100% success rate could be reached when deriving and propagating CRC organoids from different subtypes of CRC tissues. Other cohorts of cancer organoids established from esophageal cancer, metastatic CRC and gastroesophageal cancer patients also demonstrated a high degree of similarity in phenotypic and genotypic features to the original tumors.^36^^,^^58^ These results, together with other studies in which additional CRC PDOs were successfully established,^59^^,^^60^ demonstrate that this pre-clinical model can be widely adapted in the field of CRC research.

**Table 2:** PDO established in different cancer types.

	Sample number (success rate)	Patient tissue	Notes	References
**CRC**	22+4 (lost due to contamination)/27 (90%)	Resected colon tumor	Biobanking, high throughput sequencing and drug screening	Van de Wetering et al.^34^
**CRC**	10/14 (71%)	Two to four 18-gauge biopsies; Metastatic lesions	Mutations compared to patient tumor	Weeber et al.^37^
**CRC**	55 (100% for good quality samples)	Surgically resected specimens or endoscopic biopsy; Primary lesions and metastatic lesions	Niche factor for mutations	Fujii et al.^35^
**CRC**	40/63 (63%)	Biopsies; Metastatic lesions	Chemotherapy response	Ooft et al.^59^
**Gastrointestinal cancer**	110 (70%)	Biopsies; Metastasis lesions (CRC or Gastroesophageal or Cholangiocarcinoma)	Treatment response	Vlachogiannis et al.^36^
**Pancreatic cancer**	8/10 (80%)	Surgical resection	Mice pancreatic cancer organoids are also developed from KC and KPC mice	Boj et al.^61^
**Pancreatic cancer**	17/20 (85%)	Surgical resection	Pancreatic cancer modeling could also be obtained from pancreatic progenitor organoids with KRAS^G12V^ and TP53^R175H^ transduction	Huang et al.^39^
**Breast cancer**	95/155 (61%)	Breast cancer tissue or needle biopsies of metastatic breast cancer lesions	Organoids recapitulate histological and genetic features of original tumors	Sachs et al.^44^
**Lung cancer**	20/36 (55.6%)	Surgically resected lung cancer tissues and biopsy	Five histological subtypes	Kim et al.^63^
**Liver cancer**	8 successful cases	Surgical resection	Controlling the non-tumoral tissue growth by optimized isolation/culture protocol	Broutier et al.^40^
**Prostate cancer**	6/32 (15%∼20%)	Biopsy specimens and circulating tumor cells	Characterize the tumor subtypes	Gao et al.^3^
**Prostate cancer**	4/25 (16%)	Biopsy samples	Neuroendocrine prostate cancer	Puca et al.^64^
**Bladder cancer**	12/17 (70%, recent efficiency)	endoscopically by cold cup biopsy or resectoscope loop without electrical current	Study clonal evolution	Lee et al.^67^
**Glioblastomas**	Not Reported	Resected glioblastomas samples, primary and metastases	Also derived organoid from esophageal adenocarcinoma	Hubert et al.^69^
**Glioblastoma**	Not Reported	Surgical resections	Patient-derived GSCs and hESC-derived cerebral organoids.	Linkous et al.^70^
**Brain tumor**	Not Reported	Generated from hESC	Engineering on cerebral organoids. Transposon- and CRISPR–Cas9-mediated mutagenesis.	Bian et al.^76^
**Esophageal adenocarcinoma**	10/32 (31%)	Esophagectomy	Chemotherapy response	Li et al.^58^
**Endometrial cancer**	15	Hysterectomy	Chemotherapy response	Girda et al.^65^
**Endometrial cancer**	16	Biopsy samples	Drug screening	Boretto et al.^66^
**Ovarian cancer**	32/49 (65%)	Tumor resection or drainage of ascites/pleural effusion	Can be genetically modified and used for drug-screening platform	Kopper et al.^45^
**GEP-NENs**	22	Surgical resection, endoscopic biopy, or needle biopsy	Provides genetic understanding and connects the genetics and phenotypic traits	Kawasaki et al.^71^
**GEP-NENs**	5/31 (16%)	Core needle biopsies	Chemotherapy response	Dijkstra et al.^72^
**Wilms tumors**	2	From human nephroblastoma		Schutgens et al.^68^
**Head and neck squamous cell carcinoma**	8	From pathology amterial	Can be used for *in vitro* photodynamic therapy testing	Driehuis et al.^55^

Abbreviations: CRC, colorectal cancer; GEP-NENs, Gastroenteropancreatic neuroendocrine neoplasm. GSCs, glioma stem cells; hESC, human embryonic stem cell; KC, LSL-Kras^G12D/+^; Pdx1-Cre; KPC, LSL-Kras^G12D/+^; Trp53^fl/+^; Pdx1-Cre; PDO, patient-derived organoid.

PDOs for other cancer types have also been developed. Boj et al. demonstrated that organoids can be derived from surgically resected pancreatic cancer at a success rate of 80%.^61^ They further demonstrated the feasibility of deriving PDOs from fine needle aspirations, which significantly enhanced the applicability of PDO in modeling PDAC since 85% of the patients are not eligible for surgical removal of their tumors.^62^ Moreover, PDAC organoids can also be generated from PSCs after experimental transformation via lentivirally transducing the KRAS^G12V^ and TP53^R175H^ mutation, which serves as an alternative way to develop PDOs for PDAC. In addition, a large PDO biobank of breast cancer was established in 2018.^44^ This study included 155 primary and metastatic breast cancer tumors and established 95 breast cancer organoids. They demonstrated that the PDOs could recapitulate different subtypes of breast cancer that differ significantly in their histopathology, hormone receptor status and epidermal growth factor receptor 2 (HER2) status. For liver cancer organoids, their development was initially hindered because of the non-tumoral, stromal cell contamination. Certain stromal cell types from liver cancer patient specimens, such as cholangiocytes, tend to overgrow in Matrigel and become the dominant cell type. This issue was later addressed by removing R-spondin-1, Noggin and Wnt3a in the culture medium while optimizing the digestion and culturing protocol.^40^ Subsequently, organoids from common primary liver cancers (PLC), including hepatocellular carcinoma (HCC), cholangiocarcinoma (CC) and combined HCC/CC could be propagated following the new protocol. In addition, lung cancer organoids can be established from five subtypes, including lung adenocarcinoma, squamous cell carcinoma, adenosquamous carcinoma, small cell carcinoma, and large cell carcinoma.^63^ Prostate cancer organoids were first established in 2014 from biopsy specimens and circulating tumor cells, with a relatively low success rate at 15%−20%.^3^ In particular, neuroendocrine prostate cancer, a rare and aggressive prostate cancer subtype with few available pre-clinical models, has also been successfully modeled by organoids.^64^ On the other hand, PDOs can also be derived from endometrial diseases ranging from hyperplasia to low- and high-grade cancer and have been proven to maintain long-term expandability, genomic and transcriptomic stability and patient heterogeneity.^65^^,^^66^ On top of that, ovarian cancer (OC) can also be modeled by PDO. Kopper et al. established 56 organoid lines to represent all main subtypes of OC, which faithfully recapitulate the histological and genomic features of the parental tumor.^45^ Similarly, PDO can also be applied to bladder cancer studies. In 2018, 22 bladder cancer organoids were generated and found to faithfully represent tumor evolution and treatment responses of their parental tumors.^67^ Kidney cancers, the Wilms tumor, or nephroblastoma, can also be modeled by PDO.^68^ Head and neck squamous cell carcinoma (HNSCC) is another type of cancer that can be modeled by PDO and be tested with novel treatment options.^55^ Glioblastoma (GBM) or brain metastases initiated by esophageal adenocarcinoma can also be modeled by organoids.^69^ Hubert et al. revealed that GBM organoids could be directly derived from surgically removed patient tumors, and organoids derived from distinct tumor regions retained their divergent phenotypes. Importantly, brain metastases from esophageal adenocarcinoma patients, which are known to be notoriously difficult to culture *in vitro,* can be cultured as organoids.^69^ Other methods of establishing brain organoids have also been reported. For example, co-culturing the patient-derived glioma stem cells (GSCs) with human embryonic stem cell (hESC)-derived cerebral organoids demonstrated that GSCs could deeply invade the human cerebral organoid, forming tumors that phenotypically recapitulate patient GBMs.^70^ PDO can be further applied to model rare cancer types such as the gastroenteropancreatic (GEP) neuroendocrine neoplasm (NEN). Independent cohorts of GEP-NEN have been established to lay the foundation for understanding and developing therapeutics for this orphan disease.^71^^,^^72^

Most protocols for tumor organoid establishment rely on tumor tissues to be freshly retrieved from patients, but other surrogate or indirect methods are available should the direct method fail. In 2014, a single ALI culture method of organoids was developed to model the oncogenic transformation process of multiple cancer types. Recapitulating the clinical observations, pancreatic or gastric organoids bearing *Kras^G12D^* mutant gene and *p53* loss were seen to form adenocarcinoma as xenografts, while primary colon organoids need combinatorial *Apc, p53, Kras^G12D^* and *Smad4* mutations to gain tumorigenicity.^73^ Later, Hans Clevers^74^ and Toshiro Sato^75^ groups used CRISPR-Cas9 to introduce oncogenic mutations into human intestinal organoids and intestinal stem cells. All of these engineered organoids showed tumor-like features after inoculation into immunodeficient mice. Brain tumor organoids can also be established by genetically engineering normal cerebral organoids, which were transformed to glioblastoma-like or central nervous system primitive neuroectodermal tumor (CNS-PNET)-like neoplasms.^76^ Moreover, similar methods could also be applied to iPSC-initiated organoids.^77^ The iPSC-derived CRC organoid model has been used for testing therapeutic options such as chemotherapy. In addition, it could also be applied to basic cancer research, such as studying the origin of certain mutations.^78^ However, it remains unknown whether these organoids recapitulate the original tumor complexity as those PDOs derived from patients via the direct method.

Most organoids are cultured in the synthetic medium with defined molecular and growth factors. Except for the common growth factors such as EGF, which may act to inhibit cellular senescence,^79^^,^^80^ some other factors like Noggin (BMP antagonist), R-Spondin (Wnt inducer), A83–01 (TGF-β inhibitor), SB202190 (p38 inhibitor) are also required for most organoid developments. On top of this, additional specific factors may be added to the growth medium based on different cancer types. For example, Neuregulin 1, a ligand of human EGF receptor tyrosine kinases involved in mammary development and tumorigenesis, was shown to allow efficient generation of breast cancer organoids and sustain long-term expansion for > 20 passages.^44^ Individual fibroblast growth factor (FGF) family ligands contribute differently to lung organoid formation. For instance FGF7 and FGF10 induced more organoid branching as compared to FGF2 and FGF9.^81^ Moreover, the higher concentration (1 nM) of dihydrotestosterone (DHT) is important in maintaining the viability for specific types of prostate cancer organoids.^82^ We have summarized the composition of growth media for culturing PDOs of most common tumor types in Table 3. Nevertheless, further optimization for medium composition based on cancer types, stages, genetic mutations, metastatic loci and other potential factors is required to enhance the success rate of PDO establishment.

**Table 3:** A summary for PDO culture media in different cancer types.

	**Colorectal cancer**	**Gastrointestinal cancer**	**Pancreatic cancer**	**Breast cancer**	**Lung cancer**	**Liver cancer**	**Prostate cancer**	**Bladder cancer**	**Glioblastoma**
**Base**	Advanced DMEM/F12	Advanced DMEM/F12	Advanced DMEM/F12	Advanced DMEM/F12	DMEM/F12	Advanced DMEM/F12	Advanced DMEM/F12	Hepatocyte media	Neurobasalmedium
**HEPES**	10 mM		1X	10 mM		10 mM	10 mM		
**Glutamax/L-glutamine**	10 mM	2 mM	1X	1X		1%	2 mM	1X	added
**Penicillin/Streptomycin**	1X	100 unit/mL	1X	100 unit/mL	1%	1%			
**Wnt**	50% conditional medium	100 ng/mL	50% conditional medium						
**R-Spondin**	20% conditional medium	500 ng/mL	10% conditional medium	10% conditional medium			5% conditional medium		
**Noggin**	10% conditional medium	100 ng/mL	10% conditional medium	100 ng/ml			10% conditional medium		
**EGF**	50 ng/mL	50 ng/mL	50 ng/mL	5 ng/ml	50 ng/mL	50 ng/mL	50 ng/mL	10 ng/ml	added
**B27**	1X	1X	1X		added	1:50	1X		added
**n-Acetyl Cysteine**	1.25 mM		1 mM	1.25 mM		1.25 mM	1.25 mM		
**Nicotinamide**	10 mM	4 mM	10 mM	5 mM		10 mM	10 mM		
**Gastrin**	10 nM	10 nM	10 nM			10 nM			
**A83–01**	500 nM	500 nM	500 nM	500 nM		5 µM	500 nM		
**SB202190**	3 µM	5 µM		500 nM			10 µM		
**Prostaglandin E2**	10 nM	1 µM							
**Primocin**	100 µg/mL		1 mg/mL	50 µg/ml			1:100	100 µg/ml	
**Y-27,632**		10 µM		5 µM	10 µM	10 µM	10 µM	10 µM	
**N2 addiive**		1X			added	1:100			
**BSA**		0.01%							
**Basic fibroblast growth factor (bFGF)**					20 ng/mL				
**FGF-2**									
**FGF-7**				5 ng/ml			1 ng/mL		
**FGF-10**		10 ng/Ml	100 ng/ml	20 ng/ml		100 ng/ml	10 ng/ml		
**FGF-Basic**		10 ng/mL							added
**HGF**		20 ng/mL (For cholangiocarcinoma organoids only)				25 ng/mL			
**Forskolin**						10 µM			
**Dexamethasone**						3 nM			
**Neuregulin 1**				5 nM					
**Dihydrotestosterone (DHT)**							0.1–1 nM		
**Charcoal-stripped fetal bovine serum**								5%	
**sodium pyruvate**									added
**References**	Van de Wetering et al.^34^; Weeber et al.^37^	Vlachogiannis et al.^36^	Boj et al.^61^	Sachs et al.^44^	Kim et al.^63^	Broutier et al.^40^	Gao et al.^3^	Lee et al.^67^	Hubert et al.^69^

Abbreviation: PDO, patient-derived organoid.

## 3 Advanced models of PDO

PDOs have been shown to maintain the histopathological, transcriptomic and genetic characteristics of the parental tumors. However, the traditional PDOs tend to contain only cancer cells and lack components of the TME, such as fibroblasts, endothelial cells, and immune cells among others. Therefore, there are ongoing efforts focusing on adding the TME components to the organoids to recapitulate the microenvironment of the parental tumors.

For example, CAF are a critical component of TME, which play a crucial role in tumorigenesis and progression by remodeling the extracellular matrix (ECM) and producing extensive cytokines and chemokines to interact with cancer cells and infiltrating immune cells.^83^ Moreover, CAFs may induce drug resistance in tumors by either producing certain secretory molecules that protect cancer cells from drug-induced cell death or depositing matrix components that provide survival signals to cancer cells via integrins.^84^ Meanwhile, the immune system is another crucial component of TME. Advances in T cell-based immunotherapy, including the adoptive T cell transfer^85^^,^^86^ and immune-checkpoint inhibition, have revolutionized the treatment options for multiple cancer types and been proven of significant clinical benefits in patients with several types of late-stage solid tumors, including microsatellite instable (MSI) CRC, melanoma and non-small-cell lung cancer (NSCLC).^87^, ^88^, ^89^ Nonetheless, poor responses to immunotherapy are not uncommon in the clinic, which can be attributed to a variety of mechanisms. Therefore, several organoid culturing systems have been developed to co-culture organoids with CAFs and/or immune cells. These more advanced PDO models could 1) be deployed as a model to study the interactions between cancer cells and key components of the TME and 2) accurately predict a patients's sensitivity to chemotherapy and immunotherapy.

### 3.1 Co-culture models of PDO

#### 3.1.1 PDO co-culture with CAF

Reconstituting the TME by adding the CAFs to PDO models is one approach to address the need for studying interactions between cancer cells and the TME,^49^^,^^90^ especially for cancers like PDAC, which normally includes up to 90% of stroma component in the tumor mass.^91^ When studying the inter-tumor heterogeneity of PDAC based on their stem cell niche factor dependencies, Seino et al. revealed a Wnt-non-producing subtype of PDAC that requires Wnt from CAFs,^90^ which demonstrated the necessity to introduce the matched CAF into organoids culture for this subtype. Meanwhile, pancreatic stellate cells (PSCs) were shown to differentiate into αSMA-expressing CAFs when co-cultured with PDAC-PDOs.^92^ Interestingly, in addition to the αSMA-high and myofibroblasts-like CAFs, PSCs were found to differentiate into another CAF subtype named inflammatory CAFs using the co-culture platform of PSCs and PDAC-PDOs. The inflammatory CAFs in turn support tumor cell survival and growth through paracrine signaling via IL-6, IL-11 and LIF.^92^ Moreover, CAFs could also contribute to the malignant progression of CRC.^93^ Luo et al. successfully established a CRC PDO—CAFs co-culture system by embedding both cell types in 3D hyaluronan (HA)-gelatin hydrogel, which is believed to recapitulate the *in vivo* CRC ECM.^94^ Further characterization of this system demonstrated that the CRC PDO—CAFs co-culture promoted PDO growth, recovered biological pathways that are absent in the conventional PDO culture but present in patient tissues, and could be used as a platform for predicting drug responses. These studies demonstrate the great potential of PDO-based models in studying the symbiotic interactions between cancer cells and the TME components.

#### 3.1.2 PDO co-culture with PBMC derived T cells

Another important feature that is missing in the conventional PDO model is the immune components. A recently established PDO co-culture system allows the expansion and selection of tumor-reactive T cells from peripheral blood for predicting their anti-cancer properties.^51^ In this study, mismatch repair-deficient (dMMR)/MSI CRC organoids that are major histocompatibility complex (MHC) class I proficient were first developed. Then autologous PBMCs were isolated from the matched patients and cultured together with the corresponding tumor organoids. The co-culture involves pre-treatment of PDOs with Interferon (IFN)γ to enhance antigen presentation, and plate-bound anti-CD28 and IL-2 to provide co-stimulation and to support T cell proliferation. Increased tumor-reactive CD8^+^ T cell populations were detected in 50% (4/8) of the co-culture models and notably, T cell-organoid co-culture could expand previously undetectable tumor-reactive T cell populations. Co-culture of PBMCs and autologous tumor organoids could also be used in NSCLC to expand tumor-reactive CD8^+^ T cells.^51^ These autologous tumor-reactive CD8^+^ T cells were seen to substantially reduce the survival of the matched PDOs, indicating the potential of this PDO co-culture model in evaluating the responses of immuno-oncology therapies in a personalized manner and in generating patient-specific tumor-killing T cells. It should be noted that although the enriched CD8^+^ T cells were largely tumor specific, a group of expanded CD4^+^ T cells was found to recognize the murine original culture matrix (Geltrex), which was used for organoid culture in this model. To address this issue, synthetic matrices might be more appropriate in future T cell-organoid co-culture systems to avoid the possible T cell cross-reactivity to non-tumor antigens.^95^ Admittedly, deriving tumor-reactive T cells directly from tumor-infiltrating lymphocyte (TIL) can be more straightforward and less time-consuming,^96^ but the absolute number of TILs may not be sufficient in sparsely infiltrated “cold-tumors”. Therefore, deriving tumor-reactive T cells from peripheral blood permits broader applications, especially when PDOs could be successfully established from FNB.

  a) *ALI as a holistic model for studying immune component in organoids*

Besides the submerged Matrigel embedded organoids, another major form of organoid culture adopts the ALI, which was first deployed in culturing murine organoids.^73^ In this approach, PDOs grow in the collagen gel in the inner Transwell dish, which is exposed to air via the ALI to have sufficient oxygen supply, while the culture medium in the outer dish can diffuse into the collagen gel via the permeable Transwell. Using this system, diverse gastrointestinal cancer organoids were established by transforming primary organoids from the mouse colon, stomach and pancreas. Extending this method, Neal and Li et al. successfully cultured PDOs from surgically resected primary and metastatic tumors.^50^ This method was reported to show an overall 73% success rate and the established PDOs could be robustly cryorecovered. One notable advantage of this system is that it preserves the stromal components from the original normal or cancerous tissue specimens without the need of reconstitution, though the stromal cells are gradually lost over passaging. It is worth noting that in this system, CD4^+^ and CD8^+^ TILs were found to be at a substantial amount at day seven and could be sustained for more than one month when IL-2 was added. These T cells preserve the intratumoral T cell receptor (TCR) repertoire of the parental tumor. CD11b^+^ tumor-associated macrophages (TAMs) were also observed in the co-culture system at day seven along with other immune cells, including B cells and NK cells. It should be noted that recent studies revealed that a substantial fraction of TILs does not exhibit tumor-reactive properties.^97^^,^^98^ Therefore, it remains to be seen whether tumor-reactive T cells from PBMCs or the TILs from tumor tissues provide a better model in predicting the efficacy of immunotherapy in personalized medicine. Nevertheless, the diverse types of PDOs and PDO-derived co-culture models hold great promise in addressing these issues and in providing faithful *in vitro* models for immuno-oncology studies.

  b) *Microfluidic organotypic spheroids as a holistic model for studying immune component in organoids*

Despite the various advantages of PDOs in modeling tumor progression and the tumor microenvironment as mentioned above, there remains a few technical challenges associated with the use of Matrigel in organoid culture, including the heterogeneous nature of organoids' size and shape, and the limited access to the lumen space of organoids for other cell types or reagents. On the other hand, another *in vitro* cell/tissue culture system named microfluidic organ-on-chips, in which organotypic spheroid grows in 3D microfluidic culture units, could provide precise control of certain cellular, chemical and biophysical parameters and address some of these difficulties.^99^ In a typical setting of microfluidic organ-on-chips, normal tissue or tumor spheroids grow in the center of the microfluidic device, which are embedded in cell-supporting 3D gel and supplemented by media flow in the microfluidic channels. Advanced devices also permit co-culture with stroma cells to create a complex interface that mimics the orthotopic tumor microenvironment.^100^, ^101^, ^102^ MDOTs/PDOTs from syngeneic mouse models and patient tumors preserved the autologous lymphoid and myeloid cells.^103^ MDOT and PDOT were reported to preserve heterogeneity in culture and could respond to immune checkpoint blockade (ICB) treatments, allowing this method to be deployed to study combinatorial immunotherapies. Although MDOTs and PDOTs can be developed from diverse cancer types, including melanoma, Merkel cell carcinoma and CRC, this application is also confined by several limitations. For example, MDOTs/PDOTs can only be maintained in short-term culture for around 1–2 weeks, and PDOTs tend to have low reproducibility due to the issue of tumor heterogeneity. Moreover, due to the resource-intensive nature of MDOTs and PDOTs, applying high-throughput therapeutic screening is challenging on these devices.^104^

In addition, droplet-based patient-derived micro-organospheres (MOS) have been demonstrated to have the potential for high-throughput screening.^33^ The principle of this method involves a benchtop machine for generating the MOS, where dissociated cells from primary tissues were added to the Matrigel in a microfluidic chip. Then, the cell-containing Matrigel is mixed with a biphasic liquid oil to generate the droplet MOS, which is directly dispensed into a MOS recovery vessel. A prospective clinical study was conducted on this system involving eight CRC patients. On average, this system only requires about ten days to complete a high-throughput drug screening, and the drug-screening results reliably predict the clinical outcome. In addition, immune cells could be well preserved in this system and remain functional, including CD4^+^, CD8^+^ T cells, CD56^+^ Natural Killer cells and CD11b^+^ cells. Therefore, this system shows great potential for testing immune-oncology therapies, including programmed cell death-1 (PD-1)/PD-ligand 1 (PD-L1) blockade and T-cell therapies for cancer patients within a time frame that is suitable for guiding cancer treatment in the clinic. Both the microfluidic organ-on-chips and the MOS technologies are compatible with PDO models to some extent. It is interesting to see in the future how PDO models could be further improved by incorporating these technologies in order to better recapitulate the tumor microenvironment in patients.

## 4 Current applications of PDO

### 4.1 Applying PDO for precision medicine

Biomarker-directed selection of therapies in many cancer types has been demonstrated to significantly improve patient survival. For example, dMMR and high microsatellite instability (MSI-H) define a subset of CRC patients who are suitable for anti-PD-1/PD-L1 immune checkpoint blockade therapy.^105^ In addition, hormone receptors and HER2 expression status guide the application of hormone therapy and HER2-targeted therapy respectively in breast cancer.^106^ However, conventional biomarkers fail to differentiate rare subpopulations of patients, and both single and “omics”-based biomarkers often suffer for their low sensitivity and/or specificity.^107^ Therefore, there is an urgent clinical need for personalized treatment plans that can be developed based on an accurate and timely prediction by pre-clinical models. PDO shows obvious advantages in precision medicine due to its high success rate of establishment, relatively short turnaround time of the procedure and accurate prediction of sensitivity to therapies in corresponding patients. As discussed earlier, PDOs can be generated from limited starting tumor material, such as that from fine needle aspiration. Compared to PDX models that commonly take months to develop, PDOs can be established in weeks or even days utilizing some new methods. As a result, it is possible for patients to get timely treatment advice based on the drug screening results using PDO models derived from the same patient. As summarized below and in Table 4, many studies have validated the accuracy of using PDO as the drug screening model.

**Table 4:** PDO applied in therapeutic screening in different cancer types.

Tumor type	Size of cohort	Purpose	Treatment	PDO model	References
**Chemotherapy**					
mCRC	35 PDO from 29 patients	Prospective study	*N* = 16, FOLFOX → response cannot be predicted. *N* = 12, FOLFIR → response can be predicted. *N* = 10, Irinotecan → response can be predicted	Submerged 3D Matrigel	Ooft et al.^59^
mCRC (MSS, peritoneal mets)	17 PDO from 15 patients	Prospective study. Personal treatment options	*N* = 17, medium throughput: 35 drugs. *N* = 9, FOLFOX, FOLFIR	Submerged 3D Matrigel	Narasimhan et al.^108^
mCRC, mGOC	19 PDOs from 15 patients	Prospective study, clinical correlation	*N* = 19, medium throughput: 55 drugs, Paclitaxel, Regorafenib, TAS-102, Cetuximab, VX-970	Submerged 3D Matrigel	Vlachogiannis et al.^36^
Breast cancer, Pancreatic Cancer	14 PDOs (pancreatic), 13 PDOs (breast)	Observational study	Gemcitabine + 5-FU, Oxaliplatin + 5-FU, 5-FU or FOLFIRINOX (pancreatic cancer), A + C + T (breast cancer)	Submerged 3D Matrigel	Sharick et al.^109^
Pancreatic cancer	66 PDOs	Observational study, clinical correlation	Chemo: Gemcitabine, Paclitaxel, Irinotecan, 5-FU, OxaliplatinTargeted: 21 drugsTargeted for Chem-refractory: 27 drugs	Submerged 3D Matrigel	Tiriac et al.^110^
Ovarian cancer	7 PDOs from 5 patients (clinical correlation). 36 PDOs	Observational study, clinical correlation	*N* = 7, Carboplatin and Paclitaxel. *N* = 36, other 10 chemotherapy and targeted drugs	BME	de Witte et al.^111^
Breast cancer	28 PDOs for drug screening, 2 PDOs for clinical correlation	Observational study, clinical correlation	*N* = 28, Afatinib, Gefitinib, Pictilisib, GDC-0068, AZD8055, Everolimus (drug screening). *N* = 2, Afatinib	Submerged 3D Matrigel	Sachs et al.^44^
**Radiation/chemoradiation**					
RC	21 PDOs	Clinical correlation	*N* = 21, 5-FU and FOLFOX*N* = 6, Cetuximab. *N* = 19, Radiation	Submerged 3D Matrigel	Ganesh et al.^115^
RC (locally advanced)	80 PDOs from 80 Patients	Prospective and observational study	Radiation+5-FU or Radiation+Irinotecan, with neoadjuvant Capecitabine	Submerged 3D Matrigel	Yao et al.^38^
HNSCC	31 PDOs	Observational study, clinical correlation	*N* = 13, Cisplatin, Carboplatin and Cetuximab, Everolimus, AZD4547, Niraparib. *N* = 7, radiotherapy. *N* = 3, radiotherapy+LC161	BME	Driehuis et al.^116^
GBM	10 PDOs from 7 Patients	Observational study	*N* = 8, radiation + Temozolomide. *N* = 10, Gefitinib, Trametinib, Everolimus. *N* = 6, CAR T (EGFRvIII-specific)	Direct culture in suspension from fresh tumor	Jacob et al.^117^
**Immunotherapy**					
CRC (dMMR+pMMR, stage III)	12 PDOs from 12 Patients	Prospective study	Ipilimumab + Nivolumab (neoadjuvant)	Organoid+PBL	Chalabi et al.^118^
NSCLC, ccRCC, melanoma	9 NSCLS, 8 ccRCC, 3 melanoma	Observational study	Nivolumab (anti-PD-1)	ALI	Neal et al.^50^
RC	17 PDOs	Prospective study	Pembrolizumab (anti-PD-1)	PDO and TIL expanded separately	Kong et al.^96^
Bladder cancer organoids	3 PDOs	Observational study	2nd generation CAR T (MUC1-specific)	Submerged 3D Matrigel	Yu et al.^119^
ccRCC	20 PDOs	Observational study	*N* = 10, Caozantinib or Nivolumab (anti-PD-1)	ALI	Esser et al.^120^

Abbreviations: 5-FU, 5-fluorouracil; A + C + T, paclitaxel, 4-OOH cyclophosphamide and doxorubicin; ALI, air-liquid interface; BME, submerged 3D basement membrane extract; CAR, chimeric antigen receptor; ccRCC, clear cell renal cell carcinoma; Chemo, chemotherapy; dMMR, deficient DNA mismatch repair; GBM, glioblastoma; HNSCC, head and neck squamous cell carcinoma; mCRC, metastatic colorectal cancer; mGOC, metastatic gastroesophageal; MSS, microsatellite-stable; PBL, peripheral blood lymphocytes; PDO, patient-derived organoid; pMMR, proficient in mismatch repair; RC, rectal cancer; TIL, tumor infiltrating lymphocyte.

#### 4.1.1 Applying PDO for chemotherapy screening

The first attempt to use PDO for predicting treatment responses in patients was performed on a cohort of metastatic CRC (mCRC) and gastroesophageal (mGOC) patients recruited in phase 1/2 clinical trials.^36^ The results revealed a high confidence of using PDOs in predicting responses to targeted therapies and chemotherapies in patients with 100% sensitivity and 93% specificity. This study highlighted the advantage of using PDOs to predict clinical outcomes, which is better than using molecular biomarker panels alone.^36^ The follow-up TUMOROID study that focused specifically on mCRC revealed that it is clinically feasible to deploy a PDO-based screen to predict responses to irinotecan-based chemotherapy.^59^ However, this study showed conventional PDO models failed to predict responses to oxaliplatin-based chemotherapy. These findings suggest that the conventional PDO models may not fully recapitulate the cancer cell state in the parental tumor and thus have different accuracies in predicting the responses to different treatments, which in turn highlight the importance of including immune and stromal components in PDO models to rebuild the faithful tumor microenvironment. While most studies in this category were observational and summarized the correlation between clinical outcomes and PDO-based drug screening in a non-interventional manner, the APOLLO trial in 2020 was the first to offer patients the PDO-informed treatment options.^108^ This study demonstrated that medium to high-throughput drug screening on PDOs could reveal novel therapeutic options with or without validated biomarkers in addition to the standard of care. For CRC with peritoneal metastases (CRPMs), FDA-approved targeted drugs on other cancer types could be potentially repurposed to treat this poor-prognostic disease should standard care be exhausted.

While CRC remains one of the most extensively studied cancer types using PDO models, similar drug screening studies on PDOs have been applied to several other cancer types, including breast cancer,^44^^,^^109^ pancreatic cancer,^110^ and ovarian cancer.^111^ In all of these cancer types, drug responses recorded using the *in vitro* PDO models highly resemble the clinical outcomes, portending that PDO-informed treatment can lead to promising personalized oncology.

#### 4.1.2 Applying PDO for predicting responses to radiation-based therapy

In parallel to chemotherapy, many cancer types also rely on radiotherapy as the primary treatment modality, including HNSCC, rectal cancer and non-resectable glioblastoma.^112^, ^113^, ^114^ The radiation treatment on PDOs was commonly performed by placing the culture dish into the irradiator cabinet to be treated with a total dose of 1–10 Gy based on the tumor type, and then recovering by several days before measuring the cell viability. Similarly, the *in vitro* response of PDOs to radiotherapy/chemoradiation could be assessed within weeks, and the results were highly correlated with the patient responses across diverse cancer types.^38^^,^^115^, ^116^, ^117^ Although the results need to be further confirmed in larger cohorts, the promising results suggest the PDOs could be used in evaluating other treatment modalities in addition to chemotherapies.

### 4.2 Applying PDO for predicting responses to immunotherapy

The aforementioned PDO-immune cell co-culture models can be used to test the clinical response to ICI. In the NICHE study, Chalabi et al. reported that in the presence of PD-1 blocking antibodies, CRC PDOs derived from clinical responders could be recognized by autologous T cells.^118^ However, *in vitro* CD8^+^ T cell reactivity in the PDO-autologous T cell co-culture system was not seen in all clinical responders. Thus, the current platform remains to be improved to accurately predict the response to ICI therapy, though it is suitable to gain biological insights of mechanisms of resistance to ICI therapies and to develop new methods to overcome the resistance. In addition, the submerged 3D Matrigel PDO system can also be used as a platform for testing the efficacy of chimeric antigen receptor (CAR) T therapies. A recent study demonstrated that engineered CAR T cells could be co-cultured with bladder cancer organoids and induce antigen specific CAR T cell-mediated cytotoxicity.^119^ However, the accuracy of predicting CAR T cell therapy in solid tumors by PDO—CAR T co-culture platform remains to be further validated using larger patient cohorts and in more cancer types.

As mentioned above, ALI PDO culture permits the tumor cells to grow largely in their native state, retaining a significant proportion of the tumor stroma including diverse types of infiltrated immune cells. Using this method, Neal et al. demonstrated that the TILs in the NSCLC PDOs exhibited nivolumab-dependent tumor cytotoxicity, suggesting that the PD-1-dependent ICI therapy can be recapitulated by this co-culture system. A follow-up study demonstrated that for clear cell renal carcinoma (ccRCC), ALI PDO could be used to evaluate the response of combination therapy with cabozantinib and nivolumab, suggesting the potential of using ALI as a *holistic* model for precision medicine.^120^

### 4.3 Applying PDO for basic cancer research using high-throughput sequencing

Since tumor PDO can be easily biobanked and passaged at a relatively low cost compared with PDX, it is beneficial to expand precious patient tumor biopsies *in vitro* by establishing PDOs, thus enabling many downstream applications that could not be done on patient biopsies per se. For example, high-throughput sequencing for profiling genetic and epigenetic features can be easily applied to PDO, offering the possibility for a comprehensive understanding of the parental tumor. In Table 5, we summarized a list of recent publications that involved performing high-throughput sequencing on PDO. Although this list is not exhaustive, it already shows that for many cancer types, different methods of sequencing could be applied to PDO to advance our knowledge of disease progression, to improve the model development, and to provide mechanistic insights for future therapy development.

**Table 5:** PDO applied in high-throughput screening.

Tumor type	Sequencing method	Purpose of sequencing	Data accession	PDO model	References
**mCRC, mGOC**	WGS	Genomic characterization, patient stratification	European Genome-Phenome Archive (EGA) S00001002784	Submerged 3D Matrigel	Vlachogiannis et al.^36^
**CRC**	RNA-seq, WGS	Genomic, gene expression characterization, clonality study	EGAS00001003320 (RNA-seq), EGAS00001000881 (WGS)	Submerged 3D Matrigel	Roerink et al.^121^
**Pancreatic cancer**	WES, WGS, RNA-seq	Genomic, gene expression characterization, correlation with patients, patient stratification	NCBI dbGaP phs001611.v1.p1	Submerged 3D Matrigel	Tiriac et al.^110^
**Breast cancer**	WGS, RNA-seq	Genomic, gene expression characterization, correlation with patients	EGAS00001002158	Submerged 3D BME-2	Sachs et al.^44^
**Prostate cancer**	WES, RNA-seq, ERRBS	Genomic, gene expression, DNA methylation characterization	GSE112786 (RNA-seq), GSE112829 (ERRBS), SRP138000 (WES)	Submerged 3D Matrigel	Puca et al.^64^
**Prostate cancer**	WES, RNA-seq	Genomic, gene expression characterization, correlation with clinics	MSKCC cBioportal (2014 Cell)	Submerged 3D Matrigel	Gao et al.^3^
**Gastric cancer**	WES, RNA-seq	Genomic, gene expression characterization, tumor evolution	EGAS00001003145	Submerged 3D Matrigel	Yan et al.^41^
**Ovarian cancer**	WGS, sc-WGS, RNA-seq	Genomic, gene expression characterization, correlation with clinics, tumor heterogeneity, patient stratification	EGAS00001003073	Submerged 3D BME-2	Kopper et al.^45^
**CRC**	RNA-seq	Gene expression characterization	GSE77250, GSE88945, GSE82207	iPSC derived PDO	Crespo et al.^77^
**Brain tumor**	RNA-seq	Gene expression characterization of models	GSE101577, GSE110611	iPSC-derived PDO with CRISPR editing	Bian et al.^76^
**CRC**	WES, RNA-seq, microArray	Gene expression characterization of models	GSE64392, GSE65253	Submerged 3D BME-2	Van de Wetering et al.^34^
**ccRCC**	*Sc* 5′ VDJ and 5′ RNA-seq	Gene expression and T cell receptor characterization of models, drug responses	GSE111360	ALI	Neal et al.^50^
**Pancreatic cancer (metastasis)**	ATAC-seq, ChIP-seq	Disease progression, enhancer regulation	GSE99311	Submerged 3D Matrigel	Roe et al.^124^
**Breast cancer**	WES, RNA-seq, reduced representation methylation sequencing	Genomic, gene expression characterization	GSE152202, GSE186747	Submerged 3D Matrigel	Guillen et al.^130^
**PDAC**	WGS, RNA-seq, scRNA-seq	Gene expression and molecular characterization of models, cell state, effect of *ex vivo* environment on PDO	Broad Institute Single Cell Portal: SCP1644	Submerged 3D Matrigel	Raghavan et al.^122^
**Diffuse-type gastric cancer**	WES, RNA-seq	Genomic, gene expression characterization and comparison between subtypes	Not reported	Submerged 3D Matrigel	Togasaki et al.^123^

Abbreviations: ALI, air-liquid interface; ATAC, assay for transposase-accessible chromatin; BME, submerged 3D basement membrane extract; ccRCC, clear cell renal cell carcinoma; ChIP, chromatin immunoprecipitation; ERRBS, enhanced reduced representation bisulfite sequencing; iPSC, induced pluripotent stem cells; mCRC, metastatic colorectal cancer; mGOC, metastatic gastroesophageal; PDAC, pancreatic ductal adenocarcinoma; PDO, patient-derived organoid; *Sc*, single cell; WES, whole-exome sequencing; WGS, whole-genome sequencing.

Genetic profiling using whole-exome or whole-genome sequencing provides information on copy number variation (CNV), single nucleotide polymorphism (SNP) and insertion or deletion (INDELs), which is widely applied in PDO based research to ensure the faithful representation of the parental tumor. Moreover, assessing the genomic landscapes of clonal organoids allows for inferring the phylogenic tree.^51^^,^^121^ Genetic mutations in the subclones could vary as a result of clonal evolution during PDO passage, resembling the genetic clonal evolution process in human cancers *in vivo*.*^51^*

Furthermore, applying transcriptomic profiling by RNA sequencing (RNA-seq) and single-cell RNA-seq (scRNA-seq) on PDO not only allows for assessing the similarity between PDOs and the parental tumor,^122^ but also allows for investigating the inherent tumor heterogeneity. For example, Togasaki et al. applied organoid models to demonstrate that the two often-coexisting subtypes of diffuse-type gastric cancer (GC), including the signet-ring cell carcinoma (SRCC) and non-SRCC subtypes, were actually clonally identical.^123^ This was achieved by performing RNA-seq on GC organoids representing these two subtypes, which identified Wnt signaling as the key regulator of their morphological differences. In addition, single-cell level analysis on PDOs and their matched tissue samples enabled the identification of specific cancer cell states and their drivers. For example, by performing scRNA-seq on matched PDAC tumor tissue and organoids, Raghavan et al. demonstrated a selectively enriched scBasal state with transcriptional evolution in the *in vitro* PDOs compared with the parental tumors.^122^ Moreover, the culture media formulation was found to alter the transcriptional states of PDO cancer cells and their drug sensitivity, suggesting that it is essential to use parental tumors as a benchmark and correct the culture-related biases on cancer cell state in order to establish a faithful pre-clinical cancer model.

Epigenetic profiling methods can also be applied to PDO for mechanistic studies. By performing assay for transposase-accessible chromatin with high-throughput sequencing (ATAC-seq) and chromatin immunoprecipitation followed by sequencing (ChIP-seq) on PDAC organoids, Roe et al. identified an enhancer reprogramming process that could promote PDAC metastasis.^124^ By comparing the epigenome of matched primary and metastatic organoids, a more active state in metastatic organoids was revealed, and the pioneer factor FOXA1 was identified as the driver for this metastasis-promoting enhancer reprogramming. Furthermore, examining the epigenetic and transcriptomic profiling on organoids before and after certain drug treatments may reveal resistance mechanisms. For example, Tung et al. performed the integrated profiling of ATAC-seq and RNA-seq on CRC PDOs treated with the standard-of-care therapy oxaliplatin.^125^ They demonstrated that the oxaliplatin-resistant tumor cells underwent patient-specific transcriptomic and chromatin alterations to increase the FGFR1 and OXTR. Silencing both genes via CRISPR/Cas9 reversed the phenotype of oxaliplatin resistance.

## 5 PDOX - a potential substitute for PDX?

PDOXs can be derived by directly injecting PDOs into the immune-compromised mice. Similar to the conventional PDX, PDOX preserves many parental tumor features, including the histopathological traits of disease progression, drug sensitivity and tumor invasiveness, which were demonstrated in multiple cancer types, including prostate cancer, CRC, glioblastoma, HNSCC, and PDAC.^3^^,^^35^^,^^44^^,^^61^^,^^116^^,^^117^ As summarized in Table 6, in previous studies, PDOXs were mainly used to validate the *in vitro* observations^1^ for cancer cell invasiveness,^69^^,^^115^^,^^117^ metastatic potential,^40^^,^^126^ niche factor requirements,^35^ drug sensitivities,^44^^,^^51^ and mutation profiles.^117^

**Table 6:** PDOX established in different cancer types.

Cancer type	Purpose for establishing PDOX	PDOX model	Success rate	Reference
**Prostate cancer**	Validate histological and immunohistological patterns, drug response	Subcutaneous injection into SCID mice	86% (6/7)	Gao et al.^3^
**CRC**	Validate the histology and morphology of parental tumor, test for niche factor dependency *in vivo*	Xenotransplanted into the kidney subcapsules of NOG mice	Not Reported	Fujii et al.^35^
**Breast cancer**	Drug response	Orthotopic xenotransplant into the mammary fat pads of NMRI-nude mice	Not Reported	Sachs et al.^44^
**PDAC**	Probe the molecular and cellular properties of neoplastic progression of PDAC, transcriptional and proteomic analysis of genes and pathways involved in the tumor progression	Orthotopic xenotransplant in nu/nu mice	91.7%: mice tumor in nu/nu mice; 85%: mice tumor in C57Bl/6; 75% (9/12): human tumor	Boj et al.^61^
**HNSCC**	Validate the tumor origin of organoids, assess tumorigenic potential	Subcutaneous injection into NSG mice	100% (3/3)	Drieuhuis et al.^116^
**GBM**	Assess tumor cell infiltration and growth, assess drug response	Orthotopic xenotransplant into immunodeficient mice	100% (8/8)	Jacob et al.^117^
**GBM**	Assess tumor invasion *in vivo*	Orthotopic xenotransplant into NSG mice	Not Reported	Hubert et al.^69^
**Rectal cancer**	Assess tumor progression, histopathological and metastatic characteristics, and drug responses of organoids *in vivo*	Orthotopic xenotransplant into NSG mice	Not Reported	Ganesh et al.^115^
**Liver cancer**	Assess histological, growth and metastatic potential	Subcutaneous injection and kidney capsules injection into NSG mice	100% (4/4)	Broutier et al.^40^
**CRC**	Develop orthotopic transplantation system	Orthotopic xenotransplant into NSG mice	100%	Roper et al.^126^
**Ovarian cancer**	Assess the tumor invasion and histopathological characteristics and drug sensitivity	Orthotopic or subcutaneous injection into NSG mice	66.25% (53/80): subcutaneous; 57.1% (12/21): orthotopic	Kopper et al.^45^
**GEP-NEN**	Molecular and histological characterization	Injection into the kidney capsule	Not Reported	Kawasaki et al.^71^
**Bladder cancer**	Validate drug response, study clonal evolution	Orthotopic xenotransplant into NOG mice	83% (15/18)	Lee et al.^67^

Abbreviations: CRC, colorectal cancer; GBM, glioblastoma; GEP-NEN, gastroenteropancreatic-neuroendocrine neoplasm; HNSCC, head and neck squamous cell carcinoma; NOG, non-obese diabetic/Shi-scid IL-2Rγ null; NSG, NOD-SCID-*IL2rg*^−/−^; PDAC, pancreatic ductal adenocarcinoma; PDOX, patient-derived organoid-derived xenografts; SCID, severe combined immunodeficiency.

It is worth noting that PDOX enables the orthotopic transplantation of PDO, which has considerable merits since subcutaneous PDXs often fail to recapitulate tumor invasion and metastasis.^127^ Orthotopic transplantation also avoids the problems associated with conventional genetically engineered mouse models (GEMMs) of CRC, of which the high tumor burden and tumor forming in the small intestine (rather than the colorectum) have long been questioned. The orthotopically transplanted tumor can be easily modified by CRISPR-Cas9-based editing to carry oncogenic genes, which can closely recapitulate the CRC disease stage progression of adenoma-carcinoma-metastasis.^126^^,^^128^ Similarly, orthotopically transplanted breast cancer organoids were demonstrated to not only faithfully recapitulate the tumor heterogeneity but also to preserve the drug response of the original tumor.^129^ These studies highlighted the importance of selecting the tumor injection site when deriving PDOXs from PDOs.

Cancer cells can be interconverted between organoid cultures and xenografts with high efficiency. Two separate studies have shown that tumor cells underwent sequential derivation as PDOs and PDOXs maintain their major mutational spectrum and are suitable for drug screening in both bladder cancer^67^ and breast cancer.^130^ In one study, organoids were established from multiple subtypes of breast cancer PDXs. These organoids, as well as new xenografts derived from these organoids, showed high fidelity to the originating patient tumor and the initial PDXs in terms of the growth rate, proliferation pattern and histopathological characteristics.^130^ More interestingly, when studying bladder cancer-derived organoids, Lee et al. found that despite a group of organoid lines that stably maintains their phenotypes, there was a second group of organoid lines showing distinct phenotypes when cultured as PDOs but reverting to the phenotype of their parental tumors when cultured as PDOXs.^67^ Our recent work also revealed that the transcriptome and epigenome (as reflected by ATAC-seq) of PDOXs better resemble the matched PDXs and patient tumors compared with PDOs.^131^ Taken together, these results suggest that cancer cells from PDOs retain the ability to reconstitute the TME when implanted *in vivo* and through interacting with components of this cancer-cell-driven TME, to re-establish their original phenotypic cell state. In other words, culture-related deviation of cancer cell state as discussed previously may be addressed by generating PDOXs.

Based on these studies and our own results, we think that PDO and PDOX represent better pre-clinical models for cancer research and precision oncology. There are obvious advantages of this method compared with traditional 2D culture systems and the PDX models:

1) Establishing PDOs and PDOXs from patient samples show a high success rate with a reasonable temporal and monetary cost. As discussed above, the success rate of establishing PDOs from patient biopsies is much higher than that of PDXs. In many cancer types, PDOs can be established with a high success rate using FNB that contain limited patient materials, making it suitable to establish pre-clinical cancer models in broad clinical settings. In addition, numerous studies have demonstrated that xenografts deriving from organoid culture have a significantly higher success rate of establishment as compared to the xenografts directly deriving from patient samples.^35^^,^^67^^,^^117^^,^^130^ In our own hands, we observed a close to 100% success rate for PDOXs from established PDOs,^131^ a finding consistent with previous studies. Moreover, PDOs can be established at a lower cost and with a shorter time compared with PDXs; they are also easy to maintain because of their ability to be easily cryopreserved, which is highly challenging for PDX models.
2) PDOXs faithfully resemble the cancer cell state in the parental tumor and are thus suitable for examining the responses to a wide range of therapeutic agents. Compared with 2D cultured cancer cell lines, PDOs show significant improvements in maintaining the genetic and phenotypic heterogeneity of the parental tumor. However, culture-related deviation of cancer cell state in PDOs has been reported,^67^^,^^122^ which may mislead the drug screening results. On the other hand, cancer cells in PDOXs are known to revert to the original cancer cell state by reconstituting the TME. Moreover, for certain targeted therapies such as VEGF inhibitors, it is not appropriate to test in the PDO model,^132^ because the agents function through regulating the TME. In these occasions, only PDOXs can be used to evaluate the efficacy of these therapies. In summary, PDOX models hold great promise in accurately predicting the responses to a variety of therapeutic agents in their corresponding patients.
3) PDOs and PDOXs can be easily modified genetically, allowing their broad applications in basic cancer research. Similar to 2D cultured models, PDO can be easily modified genetically,^2^ and subsequently form PDOX to test the effects of these manipulations *in vivo*.*^133^* These features make PDOs and their derived PDOXs suitable for studying the biological processes of tumor initiation and progression. In fact, PDOs have been used to gain novel biological insights on both the oncogenic transformation process as well as the invasion-metastasis cascade. For example, these models were applied to study the relationships between oncogenic mutations and niche factors^90^ and to identify the epigenetic regulators that drive metastasis-promoting enhancer reprogramming.^124^

## 6 Future perspectives

Based on a retrospective study, the pooled sensitivity and specificity of PDO in predicting drug response across different tumor types were 0.81 and 0.74, suggesting that personalized tumor response to anti-cancer therapies using *ex vivo* screening on PDO could serve as a strong biomarker in predicting the clinical outcomes.^134^ However, for some rare cancer types, the success rate of establishing PDOs can go as low as 16%,^64^ suggesting that PDO-informed decision is currently not available for all patients. PDO developed from pre-treated metastases have an even lower success rate of establishment than from materials of treatment-naïve tumors. Future studies should focus on increasing the success rate of PDO establishment by optimizing the culture conditions and incorporating other advanced cell/tissue culture technologies such as the microfluidic organ-on-chips and the droplet-based patient-derived micro-organospheres.

Furthermore, it remains challenging to apply current PDO and PDOX models to examine the contributions of immune components of the TME on cancer development, which are pathologically important for disease progression. Further improvements are needed for organoids-immune cells co-culture systems and humanized mouse models so immuno-oncology studies can be faithfully performed using *in vitro* cultured models or mouse models.

**Fig. 2:**
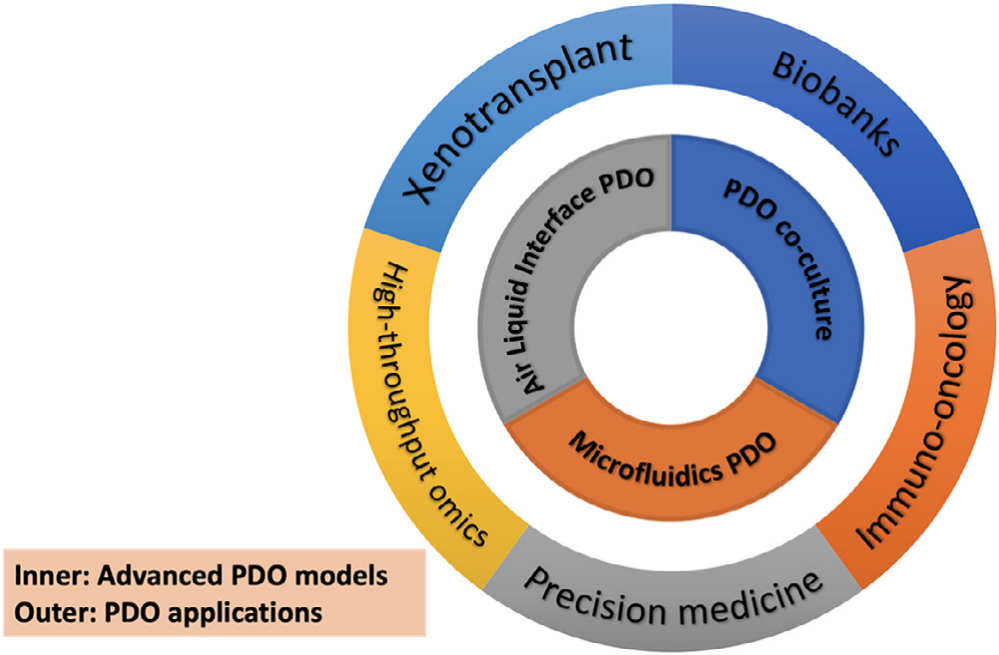
Summary of advanced models of PDO and their applications. Inner loop: advanced PDO models. Outer loop: popular applications of PDO. ECM, extracellular matrix; FNB, fine-needle biopsy; TME, tumor microenvironment.

PDOXs have been established and examined in several recent studies by injecting PDOs back into immunodeficient mice.^3^^,^^35^^,^^61^ As summarized above, this type of pre-clinical model shows several obvious advantages compared with PDX. However, more studies are needed to convincingly show PDOX still maintain essential *in vivo* features of patient tumor in different cancer types. In particular, more work should be done to determine whether the PDOX maintains the genetic and phenotypic heterogeneity of the parental tumor. If the level of heterogeneity varies in PDOX, it should be carefully examined whether such change will lead to significant differences in drug responses. Nevertheless, based on all the available experimental evidence, we believe that PDO and PDOX have a great potential to be faithful pre-clinical cancer models for both basic cancer research and precision medicine (Fig. 2).

## Declaration of competing interest

The authors declare that they have no conflict of interests.

## References

[1] Tuveson D., Clevers H. (2019). Cancer modeling meets human organoid technology. Science.

[2] Bleijs M., van de Wetering M., Clevers H., Drost J. (2019). Xenograft and organoid model systems in cancer research. EMBO J.

[3] Gao D., Vela I., Sboner A. (2014). Organoid cultures derived from patients with advanced prostate cancer. Cell.

[4] Bertotti A., Migliardi G., Galimi F. (2011). A molecularly annotated platform of patient-derived xenografts (“xenopatients”) identifies HER2 as an effective therapeutic target in cetuximab-resistant colorectal cancer. Cancer Discov.

[5] Verissimo C.S., Overmeer R.M., Ponsioen B. (2016). Targeting mutant RAS in patient-derived colorectal cancer organoids by combinatorial drug screening. Elife.

[6] Tentler J.J., Tan A.C., Weekes C.D. (2012). Patient-derived tumour xenografts as models for oncology drug development. Nat Rev Clin Oncol.

[7] Hidalgo M., Amant F., Biankin A.V. (2014). Patient-derived xenograft models: an emerging platform for translational cancer research. Cancer Discov.

[8] Kim M.P., Evans D.B., Wang H., Abbruzzese J.L., Fleming J.B., Gallick G.E. (2009). Generation of orthotopic and heterotopic human pancreatic cancer xenografts in immunodeficient mice. Nat Protoc.

[9] Marincola F., Taylor-Edwards C., Drucker B., Holder W (1987). Orthotopic and heterotopic xenotransplantation of human pancreatic cancer in nude mice. Curr Surg.

[10] Fiebig H., Neumann H., Henss H. (1985). Small cell lung cancer.

[11] Julien S., Merino-Trigo A., Lacroix L. (2012). Characterization of a large panel of patient-derived tumor Xenografts representing the clinical heterogeneity of human colorectal cancercolorectal patient-derived tumor models. Clin Cancer Res.

[12] Puig I., Chicote I., Tenbaum S.P. (2013). A personalized preclinical model to evaluate the metastatic potential of patient-derived colon cancer initiating cellsA metastasis model with patient-derived colon cancer cells. Clin Cancer Res.

[13] Marangoni E., Vincent-Salomon A., Auger N. (2007). A new model of patient tumor-derived breast cancer xenografts for preclinical assays. Clin Cancer Res.

[14] DeRose Y.S., Wang G., Lin Y.-.C. (2011). Tumor grafts derived from women with breast cancer authentically reflect tumor pathology, growth, metastasis and disease outcomes. Nat Med.

[15] Vaillant F., Merino D., Lee L. (2013). Targeting BCL-2 with the BH3 mimetic ABT-199 in estrogen receptor-positive breast cancer. Cancer Cell.

[16] Moffitt R.A., Marayati R., Flate E.L. (2015). Virtual microdissection identifies distinct tumor-and stroma-specific subtypes of pancreatic ductal adenocarcinoma. Nat Genet.

[17] Garrido-Laguna I., Uson M., Rajeshkumar N. (2011). Tumor engraftment in nude mice and enrichment in stroma-related gene pathways predict poor survival and resistance to gemcitabine in patients with pancreatic cancerpersonalized pancreatic cancer xenografts. Clin Cancer Res.

[18] Reyes G., Villanueva A., García C. (1996). Orthotopic xenografts of human pancreatic carcinomas acquire genetic aberrations during dissemination in nude mice. Cancer Res.

[19] Nguyen H.M., Vessella R.L., Morrissey C. (2017). LuCaP prostate cancer patient-derived xenografts reflect the molecular heterogeneity of advanced disease and serve as models for evaluating cancer therapeutics. Prostate.

[20] Pan C-x, Zhang H., Tepper C.G. (2015). Development and characterization of bladder cancer patient-derived xenografts for molecularly guided targeted therapy. PLoS ONE.

[21] Kim K., Hu W., Audenet F. (2020). Modeling biological and genetic diversity in upper tract urothelial carcinoma with patient derived xenografts. Nat Commun.

[22] Blomme A., Van Simaeys G., Doumont G. (2018). Murine stroma adopts a human-like metabolic phenotype in the PDX model of colorectal cancer and liver metastases. Oncogene.

[23] Bhimani J., Ball K., Stebbing J. (2020). Patient-derived xenograft models—the future of personalised cancer treatment. Br J Cancer.

[24] Roife D., Ya Kang, Wang L. (2017). Generation of patient-derived xenografts from fine needle aspirates or core needle biopsy. Surgery.

[25] Ivanics T., Bergquist J.R., Liu G. (2018). Patient-derived xenograft cryopreservation and reanimation outcomes are dependent on cryoprotectant type. Lab Invest.

[26] O'Connor N., Mulliken J., Banks-Schlegel S. (1981). Grafting of burns with cultured epithelium prepared from autologous epidermal cells. Lancet.

[27] Lindberg K., Brown M., Chaves H. (1993). *In vitro* propagation of human ocular surface epithelial cells for transplantation. Invest Ophthalmol Vis Sci.

[28] Clevers H. (2016). Modeling development and disease with organoids. Cell.

[29] Sato T., Vries R.G., Snippert H.J. (2009). Single Lgr5 stem cells build crypt-villus structures *in vitro* without a mesenchymal niche. Nature.

[30] Sato T., Stange D.E., Ferrante M. (2011). Long-term expansion of epithelial organoids from human colon, adenoma, adenocarcinoma, and Barrett's epithelium. Gastroenterology.

[31] Drost J., Clevers H. (2018). Organoids in cancer research. Nat Rev Cancer.

[32] Ootani A., Li X., Sangiorgi E. (2009). Sustained *in vitro* intestinal epithelial culture within a Wnt-dependent stem cell niche. Nat Med.

[33] Ding S., Hsu C., Wang Z. (2022). Patient-derived micro-organospheres enable clinical precision oncology. Cell Stem Cell.

[34] Van de Wetering M., Francies H.E., Francis J.M. (2015). Prospective derivation of a living organoid biobank of colorectal cancer patients. Cell.

[35] Fujii M., Shimokawa M., Date S. (2016). A colorectal tumor organoid library demonstrates progressive loss of niche factor requirements during tumorigenesis. Cell Stem Cell.

[36] Vlachogiannis G., Hedayat S., Vatsiou A. (2018). Patient-derived organoids model treatment response of metastatic gastrointestinal cancers. Science.

[37] Weeber F., van de Wetering M., Hoogstraat M. (2015). Preserved genetic diversity in organoids cultured from biopsies of human colorectal cancer metastases. Proc Natl Acad Sci U S A.

[38] Yao Y., Xu X., Yang L. (2020). Patient-derived organoids predict chemoradiation responses of locally advanced rectal cancer. Cell Stem Cell.

[39] Huang L., Holtzinger A., Jagan I. (2015). Ductal pancreatic cancer modeling and drug screening using human pluripotent stem cell–and patient-derived tumor organoids. Nat Med.

[40] Broutier L., Mastrogiovanni G., Verstegen M.M. (2017). Human primary liver cancer–derived organoid cultures for disease modeling and drug screening. Nat Med.

[41] Yan H.H., Siu H.C., Law S. (2018). A comprehensive human gastric cancer organoid biobank captures tumor subtype heterogeneity and enables therapeutic screening. Cell Stem Cell.

[42] Seidlitz T., Merker S.R., Rothe A. (2019). Human gastric cancer modelling using organoids. Gut.

[43] Beshiri M.L., Tice C.M., Tran C. (2018). A PDX/organoid biobank of advanced prostate cancers captures genomic and phenotypic heterogeneity for disease modeling and therapeutic screening. Clini Cancer Res.

[44] Sachs N., de Ligt J., Kopper O. (2018). A living biobank of breast cancer organoids captures disease heterogeneity. Cell.

[45] Kopper O., De Witte C.J., Lõhmussaar K. (2019). An organoid platform for ovarian cancer captures intra-and interpatient heterogeneity. Nat Med.

[46] Hill S.J., Decker B., Roberts E.A. (2018). Prediction of DNA repair inhibitor response in short-term patient-derived ovarian cancer organoids. Cancer Discov.

[47] Shultz L.D., Brehm M.A., Garcia-Martinez J.V. (2012). Humanized mice for immune system investigation: progress, promise and challenges. Nat Rev Immunol.

[48] De La Rochere P., Guil-Luna S., Decaudin D. (2018). Humanized mice for the study of immuno-oncology. Trends Immunol.

[49] Tsai S., McOlash L., Palen K. (2018). Development of primary human pancreatic cancer organoids, matched stromal and immune cells and 3D tumor microenvironment models. BMC Cancer.

[50] Neal J.T., Li X., Zhu J. (2018). Organoid modeling of the tumor immune microenvironment. Cell.

[51] Dijkstra K.K., Cattaneo C.M., Weeber F. (2018). Generation of tumor-reactive T cells by co-culture of peripheral blood lymphocytes and tumor organoids. Cell.

[52] Genova E., Cavion F., Lucafò M. (2019). Induced pluripotent stem cells for therapy personalization in pediatric patients: focus on drug-induced adverse events. World J Stem Cells.

[53] Yuan B., Zhao X., Wang X. (2022). Patient-derived organoids for personalized gallbladder cancer modelling and drug screening. Clin Transl Med.

[54] Driehuis E., Gracanin A., Vries R.G.J. (2020). Establishment of pancreatic organoids from normal tissue and tumors. STAR Protoc.

[55] Driehuis E., Spelier S., Beltrán Hernández I. (2019). Patient-derived head and neck cancer organoids recapitulate EGFR expression levels of respective tissues and are responsive to EGFR-targeted photodynamic therapy. J Clin Med.

[56] Kwon D.Y., Zhao Y.-.T., Lamonica J.M. (2017). Locus-specific histone deacetylation using a synthetic CRISPR-Cas9-based HDAC. Nat Commun.

[57] Jian M., Ren L., He G. (2020). A novel patient-derived organoids-based xenografts model for preclinical drug response testing in patients with colorectal liver metastases. J Transl Med.

[58] Li X., Francies H.E., Secrier M. (2018). Organoid cultures recapitulate esophageal adenocarcinoma heterogeneity providing a model for clonality studies and precision therapeutics. Nature Commun.

[59] Ooft S.N., Weeber F., Dijkstra K.K. (2019). Patient-derived organoids can predict response to chemotherapy in metastatic colorectal cancer patients. Sci Transl Med.

[60] Della Chiara G., Gervasoni F., Fakiola M. (2021). Epigenomic landscape of human colorectal cancer unveils an aberrant core of pan-cancer enhancers orchestrated by YAP/TAZ. Nat Commun.

[61] Boj S.F., Hwang C.-.I., Baker L.A. (2015). Organoid models of human and mouse ductal pancreatic cancer. Cell.

[62] Ryan D.P., Hong T.S., Bardeesy N. (2014). Pancreatic adenocarcinoma. N Engl J Med.

[63] Kim M., Mun H., Sung C.O. (2019). Patient-derived lung cancer organoids as *in vitro* cancer models for therapeutic screening. Nat Commun.

[64] Puca L., Bareja R., Prandi D. (2018). Patient derived organoids to model rare prostate cancer phenotypes. Nat Commun.

[65] Girda E., Huang E.C., Leiserowitz G.S. (2017). The use of endometrial cancer patient–derived organoid culture for drug sensitivity testing is feasible. Int J Gynecol Cancer.

[66] Boretto M., Maenhoudt N., Luo X. (2019). Patient-derived organoids from endometrial disease capture clinical heterogeneity and are amenable to drug screening. Nat Cell Biol.

[67] Lee S.H., Hu W., Matulay J.T. (2018). Tumor evolution and drug response in patient-derived organoid models of bladder cancer. Cell.

[68] Schutgens F., Rookmaaker M.B., Margaritis T. (2019). Tubuloids derived from human adult kidney and urine for personalized disease modeling. Nat Biotechnol.

[69] Hubert C.G., Rivera M., Spangler L.C. (2016). A three-dimensional organoid culture system derived from human glioblastomas recapitulates the hypoxic gradients and cancer stem cell heterogeneity of tumors found in vivobrain cancer stem cell organoids. Cancer Res.

[70] Linkous A., Balamatsias D., Snuderl M. (2019). Modeling patient-derived glioblastoma with cerebral organoids. Cell Rep.

[71] Kawasaki K., Toshimitsu K., Matano M. (2020). An organoid biobank of neuroendocrine neoplasms enables genotype-phenotype mapping. Cell.

[72] Dijkstra K.K., Van den Berg J.G., Weeber F. (2021). Patient-derived organoid models of human neuroendocrine carcinoma. Front Endocrinol (Lausanne).

[73] Li X., Nadauld L., Ootani A. (2014). Oncogenic transformation of diverse gastrointestinal tissues in primary organoid culture. Nat Med.

[74] Drost J., Van Jaarsveld R.H., Ponsioen B. (2015). Sequential cancer mutations in cultured human intestinal stem cells. Nature.

[75] Matano M., Shimokawa M., Takano A. (2015). Modeling colorectal cancer using CRISPR-Cas9–mediated engineering of human intestinal organoids. Nat Med.

[76] Bian S., Repic M., Guo Z. (2018). Genetically engineered cerebral organoids model brain tumor formation. Nat Method.s.

[77] Crespo M., Vilar E., Tsai S.-.Y. (2017). Colonic organoids derived from human induced pluripotent stem cells for modeling colorectal cancer and drug testing. Nat Med.

[78] Drost J., Van Boxtel R., Blokzijl F. (2017). Use of CRISPR-modified human stem cell organoids to study the origin of mutational signatures in cancer. Science.

[79] Yuan L., Alexander P.B., Wang X.-.F. (2020). Cellular senescence: from anti-cancer weapon to anti-aging target. Sci China Life Sci.

[80] Alexander P.B., Yuan L., Yang P. (2015). EGF promotes mammalian cell growth by suppressing cellular senescence. Cell Res.

[81] Rabata A., Fedr R., Soucek K. (2020). 3D cell culture models demonstrate a role for FGF and WNT signaling in regulation of lung epithelial cell fate and morphogenesis. Front Cell Dev Biol.

[82] Servant R., Garioni M., Vlajnic T. (2021). Prostate cancer patient-derived organoids: detailed outcome from a prospective cohort of 81 clinical specimens. J Pathol Clin Res.

[83] Sahai E., Astsaturov I., Cukierman E. (2020). A framework for advancing our understanding of cancer-associated fibroblasts. Nat Rev Cancer.

[84] Feng B., Wu J., Shen B. (2022). Cancer-associated fibroblasts and resistance to anticancer therapies: status, mechanisms, and countermeasures. Cancer Cell Int.

[85] Rosenberg S.A., Restifo N.P. (2015). Adoptive cell transfer as personalized immunotherapy for human cancer. Science.

[86] Tran E., Robbins P.F., Lu Y.-.C. (2016). T-cell transfer therapy targeting mutant KRAS in cancer. N Engl J Med.

[87] Overman M.J., McDermott R., Leach J.L. (2017). Nivolumab in patients with metastatic DNA mismatch repair-deficient or microsatellite instability-high colorectal cancer (CheckMate 142): an open-label, multicentre, phase 2 study. Lancet Oncol.

[88] Larkin J., Chiarion-Sileni V., Gonzalez R. (2015). Combined nivolumab and ipilimumab or monotherapy in untreated melanoma. N Engl J Med.

[89] Borghaei H., Paz-Ares L., Horn L. (2015). Nivolumab *versus* docetaxel in advanced nonsquamous non–small-cell lung cancer. N Engl J Med.

[90] Seino T., Kawasaki S., Shimokawa M. (2018). Human pancreatic tumor organoids reveal loss of stem cell niche factor dependence during disease progression. Cell Stem Cell.

[91] Moir J.A., Mann J., White S.A. (2015). The role of pancreatic stellate cells in pancreatic cancer. Surg Oncol.

[92] Öhlund D., Handly-Santana A., Biffi G. (2017). Distinct populations of inflammatory fibroblasts and myofibroblasts in pancreatic cancer. J Exp Med.

[93] Zhang R., Qi F., Shao S. (2019). Human colorectal cancer-derived carcinoma associated fibroblasts promote CD44-mediated adhesion of colorectal cancer cells to endothelial cells by secretion of HGF. Cancer Cell Int.

[94] Luo X., Fong E.L.S., Zhu C. (2021). Hydrogel-based colorectal cancer organoid co-culture models. Acta Biomater.

[95] Gjorevski N., Sachs N., Manfrin A. (2016). Designer matrices for intestinal stem cell and organoid culture. Nature.

[96] Kong J.C.H., Guerra G.R., Millen R.M. (2018). Tumor-infiltrating lymphocyte function predicts response to neoadjuvant chemoradiotherapy in locally advanced rectal cancer. JCO Precis Oncol.

[97] Simoni Y., Becht E., Fehlings M. (2018). Bystander CD8+ T cells are abundant and phenotypically distinct in human tumour infiltrates. Nature.

[98] Scheper W., Kelderman S., Fanchi L.F. (2019). Low and variable tumor reactivity of the intratumoral TCR repertoire in human cancers. Nat Med.

[99] Sontheimer-Phelps A., Hassell B.A., Ingber D.E. (2019). Modelling cancer in microfluidic human organs-on-chips. Nat Rev Cancer.

[100] Chen M.B., Whisler J.A., Fröse J. (2017). On-chip human microvasculature assay for visualization and quantification of tumor cell extravasation dynamics. Nat Protoc.

[101] Miller C.P., Tsuchida C., Zheng Y. (2018). A 3D human renal cell carcinoma-on-a-chip for the study of tumor angiogenesis. Neoplasia.

[102] Wu X., Newbold M.A., Gao Z. (2017). A versatile microfluidic platform for the study of cellular interactions between endothelial cells and neutrophils. Biochim Biophys Acta Gen Subj.

[103] Jenkins R.W., Aref A.R., Lizotte P.H. (2018). Ex vivo profiling of PD-1 blockade using organotypic tumor spheroids. Cancer Discov.

[104] Aref A.R., Campisi M., Ivanova E. (2018). 3D microfluidic ex vivo culture of organotypic tumor spheroids to model immune checkpoint blockade. Lab Chip.

[105] Le D.T., Uram J.N., Wang H. (2015). PD-1 blockade in tumors with mismatch-repair deficiency. N Engl J Med.

[106] Diaby V., Tawk R., Sanogo V. (2015). A review of systematic reviews of the cost-effectiveness of hormone therapy, chemotherapy, and targeted therapy for breast cancer. Breast Cancer Res Treat.

[107] Vargas A.J., Harris C.C. (2016). Biomarker development in the precision medicine era: lung cancer as a case study. Nat Rev Cancer.

[108] Narasimhan V., Wright J.A., Churchill M. (2020). Medium-throughput drug screening of patient-derived organoids from colorectal peritoneal metastases to direct personalized therapyfunctional precision medicine for advanced colorectal cancer. Clini Cancer Res.

[109] Sharick J.T., Walsh C.M., Sprackling C.M. (2020). Metabolic heterogeneity in patient tumor-derived organoids by primary site and drug treatment. Front Oncol.

[110] Tiriac H., Belleau P., Engle D.D. (2018). Organoid profiling identifies common responders to chemotherapy in pancreatic cancerpancreatic cancer organoids parallel patient response. Cancer Discov.

[111] de Witte C.J., Valle-Inclan J.E., Hami N. (2020). Patient-derived ovarian cancer organoids mimic clinical response and exhibit heterogeneous inter-and intrapatient drug responses. Cell Rep.

[112] Qian J.M., Schoenfeld J.D. (2021). Radiotherapy and immunotherapy for head and neck cancer: current evidence and challenges. Front Oncol.

[113] Roeder F., Meldolesi E., Gerum S. (2020). Recent advances in (chemo-) radiation therapy for rectal cancer: a comprehensive review. Radiat Oncol.

[114] Mann J., Ramakrishna R., Magge R. (2018). Advances in radiotherapy for glioblastoma. Front Neurol.

[115] Ganesh K., Wu C., O'Rourke K.P. (2019). A rectal cancer organoid platform to study individual responses to chemoradiation. Nat Med.

[116] Driehuis E., Kolders S., Spelier S. (2019). Oral mucosal organoids as a potential platform for personalized cancer therapyoral mucosal organoids as personalized cancer models. Cancer Discov.

[117] Jacob F., Salinas R.D., Zhang D.Y. (2020). A patient-derived glioblastoma organoid model and biobank recapitulates inter-and intra-tumoral heterogeneity. Cell.

[118] Chalabi M., Fanchi L.F., Dijkstra K.K. (2020). Neoadjuvant immunotherapy leads to pathological responses in MMR-proficient and MMR-deficient early-stage colon cancers. Nat Med.

[119] Yu L., Li Z., Mei H. (2021). Patient-derived organoids of bladder cancer recapitulate antigen expression profiles and serve as a personal evaluation model for CAR-T cells *in vitro*. Clin Transl Immunology.

[120] Esser L.K., Branchi V., Leonardelli S. (2020). Cultivation of clear cell renal cell carcinoma patient-derived organoids in an air-liquid interface system as a tool for studying individualized therapy. Front Oncol.

[121] Roerink S.F., Sasaki N., Lee-Six H. (2018). Intra-tumour diversification in colorectal cancer at the single-cell level. Nature.

[122] Raghavan S., Winter P.S., Navia A.W. (2021). Microenvironment drives cell state, plasticity, and drug response in pancreatic cancer. Cell.

[123] Togasaki K., Sugimoto S., Ohta Y. (2021). Wnt signaling shapes the histologic variation in diffuse gastric cancer. Gastroenterology.

[124] Roe J.-.S., Hwang C.-.I., Somerville T.D. (2017). Enhancer reprogramming promotes pancreatic cancer metastasis. Cell.

[125] Tung K.-.L., Chen K.-.Y., Negrete M. (2021). Integrated chromatin and transcriptomic profiling of patient-derived colon cancer organoids identifies personalized drug targets to overcome oxaliplatin resistance. Genes Dis.

[126] Roper J., Tammela T., Cetinbas N.M. (2017). *In vivo* genome editing and organoid transplantation models of colorectal cancer and metastasis. Nat Biotechnol.

[127] Lai Y., Wei X., Lin S. (2017). Current status and perspectives of patient-derived xenograft models in cancer research. J Hematol Oncol.

[128] O'Rourke K.P., Loizou E., Livshits G. (2017). Transplantation of engineered organoids enables rapid generation of metastatic mouse models of colorectal cancer. Nat Biotechnol.

[129] Duarte A.A., Gogola E., Sachs N. (2018). BRCA-deficient mouse mammary tumor organoids to study cancer-drug resistance. Nat Methods.

[130] Guillen K.P., Fujita M., Butterfield A.J. (2022). A human breast cancer-derived xenograft and organoid platform for drug discovery and precision oncology. Nat Cancer.

[131] Xiang K., Wang E., Rupprecht G., et al. Chromatin remodeling in patient-derived colorectal cancer models. *BioRxiv.* 2022. 10.1002/advs.202303379 PMC11040356 38380561

[132] Kissel M., Berndt S., Fiebig L. (2017). Antitumor effects of regorafenib and sorafenib in preclinical models of hepatocellular carcinoma. Oncotarget.

[133] Dekkers J.F., van Vliet E.J., Sachs N. (2021). Long-term culture, genetic manipulation and xenotransplantation of human normal and breast cancer organoids. Nat Protoc.

[134] Wensink G.E., Elias S.G., Mullenders J. (2021). Patient-derived organoids as a predictive biomarker for treatment response in cancer patients. NPJ Precis Oncol.

